# Correlates of Silence: Enhanced Microstructural Changes in the Uncinate Fasciculus

**DOI:** 10.3389/fpsyg.2020.543773

**Published:** 2020-10-08

**Authors:** Tal Dotan Ben-Soussan, Fabio Marson, Claudia Piervincenzi, Joseph Glicksohn, Antonio De Fano, Francesca Amenduni, Carlo C. Quattrocchi, Filippo Carducci

**Affiliations:** ^1^Research Institute for Neuroscience, Education and Didactics, Patrizio Paoletti Foundation, Assisi, Italy; ^2^Department of Physiology and Pharmacology, Neuroimaging Laboratory, Sapienza University, Rome, Italy; ^3^Human Neuroscience Department, Neuroimaging Laboratory, Sapienza University, Rome, Italy; ^4^Department of Criminology, Bar-Ilan University, Ramat Gan, Israel; ^5^The Leslie and Susan Gonda (Goldschmied) Multidisciplinary Brain Research Center, Bar-Ilan University, Ramat Gan, Israel; ^6^Department of Neuroscience, Imaging, and Clinical Science, Behavioral Imaging and Neural Dynamics (BIND) Center, University of Chieti-Pescara G. d’Annunzio, Chieti, Italy; ^7^Department of Educational Sciences, Roma Tre University, Rome, Italy; ^8^Departmental Faculty of Medicine and Surgery, Università “Campus Bio-Medico di Roma,” Rome, Italy

**Keywords:** silence, mind-wandering, attentional effort, DTI, uncinate fasciculus

## Abstract

Silence is an important aspect of various meditation practices, but little work has focused specifically on the underlying neurophysiology of silence-related meditative practice, and on how it relates to the self-reported experiences of practitioners. To expand current knowledge regarding the neurophenomenology of silence in meditation, we directly investigated first-person reports of silence-related experiences during the practice of Quadrato Motor Training (QMT) and their association with changes in fractional anisotropy (FA). Participants recorded their cognitive, emotional, and physical experiences upon beginning QMT and again after 6 weeks of QMT practice. These reports were evaluated qualitatively and quantitatively. Findings showed that change between the two time points in self-reported silence-related experiences was negatively correlated with change in attentional effort, and positively correlated with changes in the left uncinate fasciculus. These results expand current knowledge regarding the neuroanatomical correlates of silence-related experiences during meditation.

## Introduction

Silence plays a role in most Eastern and Western meditative practices, such as Zen meditation and Vipassana (Lin et al., 2008; Shonin et al., 2013). In the history of Buddhist doctrines described in Ariya Pariyesanā Sutta, for example, the Buddha urges monks to maintain “Noble Silence” (Anālayo, 2017). This expression represents a meditative state called “dhyana,” an altered state of consciousness characterized by freedom from thoughts and mental representations, enabling a deep stillness (Feuerstein, 1996).

While many researchers have emphasized the importance of silence in meditation (Vago and Zeidan, 2016; Pfeifer et al., 2019, 2020), to date little work has focused on the self-reported experiences of practitioners and the underlying neurophysiological changes associated with silence-related experience of meditative practices (Hernández et al., 2018). For example, a 15 min Quiet Time training consisting of meditation or another quiet activity such as reading silently was found to significantly increase resilience and decrease anxiety compared to a control group, showing increased emotional regulation (Wendt et al., 2015). The students who spent more time meditating also had higher resilience scores, self-reported improved sleep, happiness, and self-confidence.

It has recently been suggested that through a voluntary act of silence, used as a paradigm similar to sensory deprivation one can intentionally move from the narrative self, to the minimal and eventually overcoming the self (Paoletti et al., 2020; Paoletti and Ben-Soussan, 2020). The state of overcoming of the self, which can further be compared with self-transcendence, absorption, and non-dual states (Josipovic, 2014; Vieten et al., 2018), is characterized by changes in the perception of time and space shifting gradually toward “timelessness” and “spacelessness” (Berkovich-Ohana et al., 2013; Wittmann, 2015, 2020). Importantly, similar experiences following the Quadrato Motor Training (QMT) have been reported (Ben-Soussan et al., 2013, 2014b, 2019). For example, a recent cross-sectional study revealed that advanced-QMT practitioners showed longer and more accurate produced durations in a time production task, than did Aikido practitioners and a physically inactive control group (Ben-Soussan et al., 2019), suggesting that similarly to other forms of meditation (Wittmann, 2015), QMT has the capacity to dilate the subjective time experienced, possibly by inducing greater awareness of the present moment and of the body. In fact, QMT has been recently ascribed to mindfulness practices involving movement (Diamond and Ling, 2019), trading off speed with the opportunity for mindfulness and reflectivity (Ben-Soussan et al., 2014b; Diamond and Ling, 2017).

Mindful movements are characterized by a focus on movement in the present moment while excluding other thoughts and body movements (Kabat-Zinn, 2009; De Fano et al., 2019). Mindful movement practices involve key aspects of mindfulness such as preparation and execution of movement, regulation of attention, working memory, and decreased mind-wandering (Russell and Arcuri, 2015), mind-wandering being a construct opposing that of mindfulness (Mrazek et al., 2012). QMT involves each of these aspects: regulation of divided attention, working memory updating (e.g., noting one’s current location to know where to move to next), and prevention of mind-wandering via a need to be “in the here and now” due to constantly updating commands (Ben-Soussan et al., 2014b; De Fano et al., 2019). Mindful movement further engages “higher-order” inhibition and response selection that underlie attention and cognitive control that require moment-by-moment sensorimotor updating (Clark et al., 2015; Kimmel and Rogler, 2018). In line with this, QMT requires second-by-second mindful awareness for attending the upcoming next command (Ben-Soussan et al., 2014b; De Fano et al., 2019).

QMT (for a recent review see De Fano et al., 2019) is a movement meditation in which both individual experience and brain functioning have been examined. During QMT, practitioners step in different directions on a square delineated on the floor. The method requires smoothly executed, goal-directed behavior in response to predetermined verbal instructions separated by silent interstimulus intervals (ISIs), which are known to increase the duration of attention (Leckart et al., 1970). These quiet interstimulus intervals can be considered moments of external silence permitting also inner silence in which the participant awaits the upcoming command. In fact, it has previously been claimed that silence allows for better management of stimuli, representing a state of preparation in and toward emptiness (Teschner, 1981; Davies and Turner, 2002; Stratton, 2015; Paoletti and Ben-Soussan, 2020) which in the current context is manifested by the upcoming command.

In line with this, previous work suggests that QMT can induce a *state of waiting* (Ben-Soussan et al., 2014b), which might decrease mind-wandering and narrative-focused thought (Farb et al., 2007), thereby eliciting the experience of inner silence and increase a state of mindfulness. A month of QMT by both meditation practitioners and non-practitioners groups, in contrast to a single session of QMT was further found to report altered states of consciousness and attentional effort experiences. Crucially, these experiences were reported by the groups that practiced the QMT for 4 weeks, but not in the group that practiced it for only 1 day (Ben-Soussan et al., 2017).

In a recent longitudinal study, Piervincenzi et al. (2017) further reported that six weeks of daily QMT practice led to a bilateral increase of fractional anisotropy (FA) in tracts related to sensorimotor and cognitive functions (including the corticospinal tracts, anterior thalamic radiations, and uncinate fasciculi), reflecting better white matter integrity. However, the relationship between the brain changes and self-reported experiences associated with QMT remains currently unknown, particularly with respect to silence-related components.

Importantly, there is growing evidence that increased mindfulness, may act as a marker for improved emotion regulation skills (Creswell et al., 2007) and positive reappraisal (Garland et al., 2009, 2011). These two cognitive coping strategies are thought to be mechanisms by which mindfulness regulates emotion and stress (Vago and David, 2012), functions which are becoming increasingly important. In this context, it is important to note that the uncinate fasciculus is a tract of fibers connecting limbic structures to the prefrontal cortex. The uncinate fasciculus is associated with many functions ranging from learning visual associations to episodic memory, language and social emotional processing (Thomas et al., 2012, 2015; Von Der Heide et al., 2013). It is critical for both emotion regulation, transformation and reappraisal, and may thus underlie the improvements observed in the study of the effects of different types of meditation (Vago and David, 2012; d’Arbeloff et al., 2018).

These results related to the relationship between reappraisal, mindfulness and neurophysiological changes are further in line with different models of consciousness, such as the Self-awareness, self-regulation, and self-transcendence (S-ART) model (Vago and David, 2012) and the Sphere Model of Consciousness (SMC, Paoletti, 2002; Paoletti and Ben-Soussan, 2019, 2020; Paoletti et al., 2020). According to the SMC, reappraisal and voluntary movement toward a state of contentless consciousness, also defined as *momentary silent consciousness* (Baars, 2013; Josipovic, 2019) may have a decisive role in the experience (Paoletti et al., 2020; Paoletti and Ben-Soussan, 2020), in which focusing one’s attention on silence can be used as a paradigm for facilitating better internal communication through an internal environment intentionally created (Paoletti and Selvaggio, 2013; Paoletti et al., 2017; De Fano et al., 2019). Consequently, in this study, silence was operationalized as self-reported absence of thoughts and a subjective experience of mental quietness as suggested by various authors (Rudrauf et al., 2003; Hinterberger et al., 2014; Josipovic, 2014, 2019; Winter et al., 2019; see Table 1), thus describing a state in which participants spontaneously experienced and reported a sort of contentless consciousness.

**TABLE 1:** Definition of the macro-categories and sub-units produced by qualitative content analysis of diary entries on Quadrato Motor Training (QMT).

**Macro-category**	**Sub-unit**	**Definition**	**References**
**Macro-categories and sub-units**			
Attentional Effort	Attention and concentration	Increased focused attention and/or concentration during QMT	Coull, 1998
	Tiredness	Temporary sensation of fatigue due to QMT	Ream and Richardson, 1996; Abbiss and Laursen, 2007
	Distraction	Temporary inability to selectively focus attention on QMT-related stimuli (e.g., command, posture)	Kahneman, 1973
Mindfulness	Stability and harmony of the body	Feeling physically well, balanced, vigorous; positive body image and body schema	Ryan and Frederick, 1997; Cahn and Polich, 2006
	Positive emotion	Pleasant emotions (e.g., joy, happiness, relaxed); positive moods and/or attitudes	Cahn and Polich, 2006; Niven, 2013; Paoletti and Ben-Soussan, 2019
	“Being in the Waiting”	Ease of attending to the next instruction during QMT practice (e.g., without anticipating movements)	Ben-Soussan et al., 2017
Altered State of Consciousness	Intuition	Sudden and/or unexpected solution to a problem and/or change of perspective regarding a specific event	Wackermann et al., 2002; Kahneman, 2003
	Spontaneous visualization	Spontaneous visuospatial imagery (e.g., geometrical patterns, pictures, landscapes and/or lights)	Dane and Pratt, 2007; Lindahl et al., 2014
	Sense of wonder	Unexpected surprise, pleasure and/or enjoyment related to thoughts, feelings, and experience	Goldstein, 2002; Jacob et al., 2009
Silencing	Reduced mind-wandering	Decrease in intrusive, non-task-related, and/or irrelevant thoughts	Smallwood and Schooler, 2006; Mrazek et al., 2012; Vago and Zeidan, 2016
	Silence	Absence of thoughts; subjective experience of mental quietness	Rudrauf et al., 2003; Hinterberger et al., 2014; Josipovic, 2014, 2019; Winter et al., 2019

*The original three macro-categories were defined in Ben-Soussan et al. (2017).*

While the described neuroplastic changes are intriguing and can potentially underlie the benefits ascribed to the practice of mindfulness meditation, it has often been neglected to actually test the relationship between neuroplastic processes and changes in *lived experience* (Varela et al., 2016), especially in the context of silence. To expand current knowledge regarding the neurophenomenology of silence in meditation, we used a new approach to investigate first-person reports of silence-related experiences during QMT in relation to the changes in white matter as measured by FA. Together with the subjective experiences of silence during QMT practice, we expected to find an association between changes in silence-related self-report categories and FA changes.

Examining the relationship between silence and neuroanatomical changes can aid in unraveling the neuronal mechanisms underlying potential changes in the lived experience reported by subjects, as well as training induced-silence, and cognitive and emotional changes reported by the participants (Ben-Soussan et al., 2013, 2017). As silence, which is both required and produced in the QMT, may also be the means by which attention and mindfulness could be increased (Ben-Soussan et al., 2017; Paoletti et al., 2020; Paoletti and Ben-Soussan, 2020), and since these changes have previously been linked to, among others, FA changes in the uncinate fasciculus (Luders et al., 2011; Tang et al., 2012; Hölzel et al., 2016; Piervincenzi et al., 2017), we hypothesized that changes in self-reported silence-related experiences would be correlated with changes in the uncinate fasciculus structural connectivity. More specifically, we expected to find a positive correlation between change in these two variables, reflecting a silence-related enhanced connectivity in brain structures previously reported to be related to emotional and cognitive regulation associated with mindful experience (Creswell et al., 2007; Garland et al., 2009, 2011). In this context, it is important to note that the uncinate fasciculus is a bidirectional, long-range white matter tract that connects lateral orbitofrontal cortex and Brodmann area 10 with the anterior temporal lobes (Schmahmann and Pandya, 2007; Wheeler et al., 2017). Brodmann area 10 is thought to enable maintenance of previously running tasks in a pending state for subsequent retrieval and execution upon completion of an ongoing task (Koechlin and Hyafil, 2007) – an ability that is constantly required during QMT (Ben-Soussan et al., 2014a,b; De Fano et al., 2019). In addition, it is further related to emotional regulation (Steffens et al., 2011), which was previously found to improve following QMT (for a recent review see De Fano et al., 2019).

In addition, we examined relationships between spontaneous silence-related and other categories of self-reported experience during QMT. Silence shares characteristics with other states elicited by deliberative mindful practice, such as flow (Ben-Soussan et al., 2013) and mindfulness (Ben-Soussan et al., 2017), both of which are associated with reduced attentional effort (Harmat et al., 2015). For this reason, we further wanted to investigate possible interactions between experience of silence and other states or cognitive functions associated with QMT practice. In accordance with previous studies, we expected changes in silence-related categories to be positively correlated with change in mindfulness categories, and to be negatively correlated with change in attentional effort categories.

## Methods

### Participants and Design

We recruited 50 healthy volunteers in accordance with the inclusion and exclusion criteria reported in Piervincenzi et al. (2017). The researchers explained the study aims and procedures to participants and verified that they understood. All participants signed written informed consent forms in accordance with the Declaration of Helsinki. The ethical committee of the Università Campus Bio-Medico di Roma, Rome, Italy, approved the experimental phase I study entitled “Effect of Quadrato Motor Training on the brain of healthy volunteers” (MOTO-BRAIN, 09/14 PAR ComEt CBM). The present study was part of a larger project investigating longitudinal effects of QMT using various brain imaging techniques (results published in Lasaponara et al., 2017; Piervincenzi et al., 2017). Participants underwent magnetic resonance imaging (MRI) and electroencephalography (EEG) at two time points: the day of recruitment (*t*_0_) and following 6 weeks of daily QMT practice (*t*_1_). Participants were asked to keep a personal diary to document information about their practice and habits during the period of exercise. After excluding participants who did not complete the questionnaires at both time points, the final sample included 22 healthy right-handed participants (12 women, mean age 35.6 years, *SD* 5.4).

### Quadrato Motor Training (QMT)

During QMT, participants stand at one corner of a 0.5m × 0.5 m square and move to different corners in response to recorded verbal instructions provided at variable intervals indicating the next corner to which they should move. In order to correctly perform QMT there are different rules such as start each step with the leg inside the square (i.e., the right leg when on the left side of the square and *vice versa*), wait for the instruction without anticipating the movement and keep their gaze on an empty wall without looking at the platform or the position of the feet. So, participants must carefully control the quality of each movement, inhibit automatic motor responses before the instruction and divide their attention toward the external instructions, the proper task execution and the internal state of the body, sustaining their attention for the whole practice duration. For additional details, see Ben-Soussan et al. (2013).

### First-Person Reports

Participants kept a diary in which they were required to document their responses to three questions, regarding their (1) cognitive, (2) emotional, and (3) physical experiences during QMT. Data from the diaries were coded into an Excel worksheet and analyzed using qualitative content analysis (Mayring, 2004). In the first step, we constructed a grid to define the content categories. We began this process with a grid developed and validated in previous work (Ben-Soussan et al., 2017) and proceeded describing macro-categories and sub-units that will be considered to evaluate the content of first person reports. Along with these already used categories, we added an additional category related to silence and reduced mind-wandering (collectively called “Silencing”), which represents the main focus of the current study. We included Reduced Mind-wandering in the Silencing macro-category, as the reduction in intrusive, irrelevant, and unintentional thoughts represents an important step toward quiet restful presence (Vago and Zeidan, 2016). An exhaustive list of macro-categories and sub-units with their definitions is provided in Table 1.

In this study, response provided to each of the three questions served as the meaningful unit of analysis. Our corpus thus comprised 132 units of analysis, which could be coded with one or more indicators depending on the content (examples of coding for each sub-unit are provided in Table 2). This codification method was chosen because it enables occurrence and co-occurrence analyses (see Qualitative Analysis section below). Three researchers coded the answers using Atlas.ti software (Atlas.ti Scientific Software Development GmbH, Berlin, Germany). Inter-coder reliability was calculated using Krippendorff’s alpha, an index based on disagreements rather than agreements in the coding phase. According to Krippendorff (2018), the simplest inter-coder agreement coefficient is:

**TABLE 2:** Examples of reports categorized in each of the content analysis sub-units.

**Macro-category**	**Sub-unit**	**Example**
**Examples of quotations for each sub-unit**		
Attentional effort	Attention and concentration	*Less dispersion, my reasoning is slower but more focused* (S31).
	Tiredness	*Tired and drowsy*. I need to be stimulated to activate myself (S39).
	Distraction	“In a hurry” *between thoughts of “it’s late,” “I have to do thousands of things”* and thoughts of “stay here, you have all the time” (S16).
Mindfulness	Stability and harmony of the body	*I am feeling looser, light, stronger, and more energetic.* I haven’t had a cold yet (S34).
	Positive emotion	I like it. I feel in relation with space. *I feel peace* and a quiet curiosity (S25).
	“Being in the waiting”	I felt full willingness to perceive the experience as *being able to be in the waiting without expectations or anxiety* (S01).
Altered state of consciousness	Intuition	Thought fluidity, *association of thoughts in a “creative” and resolving way (= solutions), intuitiveness* (S11).
	Spontaneous visualization	Even during the day, sometimes *I visualize the square* (S18).
	Sense of wonder	Happy, *enthusiastic*, nervous, *surprised by the speed of time passed during the exercise* (S40).
Silencing	Reduced mind-wandering	Increased perception of the space around me, *reduced number of repetitive thoughts* (S18).
	Silence	An increased *emptiness of thoughts*, even in the part of the sequence that I know, I am able to wait for the next instruction. Time is dilated. Attention is expanded. *Silence* (S25).

*The relevant parts related to the sub-unit are marked in italics*.

$$\alpha =1-\frac{D_{o}\,\text{observed}\,\text{disagreement}}{D_{e}\,\text{expected}\,\text{disagreement}}$$

The acceptability threshold for this index is >0.667, whereas >0.800 is considered good (Krippendorff, 2018). We calculated Krippendorff’s alpha separately for each macro-category used in the study, resulting in an overall alpha of 0.758 (Silencing: 0.906, Attentional Effort: 0.728, Mindfulness: 0.797, Altered State of Consciousness: 0.388). Given the low reliability of the Altered State of Consciousness category, we excluded it from further analysis.

### Qualitative Analysis

Occurrence analysis was used to determine how many times a specific category was assigned to a sentence in *t*_0_ and *t*_1_ time points. This kind of analysis allows comparison of each macro-category and sub-unit occurrence between time points (taking all participants into account). Co-occurrence defines how many times two sub-units are coded together in the same sentence. Two main measures were utilized: the number of times a specific co-occurrence between two units was found and the strength of the relationship between two units (c-coefficient). C-coefficient varies between 0 (no co-occurrence) and 1 (full co-occurrence). It is calculated as follows:

c-coefficient=n/12(n+1n)2-n12

where *n*_12_ is the co-occurrence frequency of two codes, while *n*_1_ and *n*_2_ are their occurrence frequency.

### Quantitative Analysis

To conduct quantitative and correlation analyses between the first person reports and DTI data, we quantified the scores of the macro-categories and sub-units for each participant. As indicated above, each participant answered three questions. We assigned one point to a sub-unit every time it was coded in one of the answers. Thus, the score range for each sub-unit was from 0 (never coded) to 3 (coded in each answer). Finally, we grouped the scores based on macro-category (Ben-Soussan et al., 2017; see Table 1). In this way, we obtained quantified scores that represented the salience of each macro-category for each participant. We then calculated a delta score by subtracting *t*_0_ scores from *t*_1_ scores. A positive delta indicates increased reference to a category from *t*_0_ to *t*_1_ while a negative delta indicates a decrease between the two time points.

All quantitative analyses were conducted using STATISTICA 10 (StatSoft Inc., Tulsa, United States). We compared *t*_0_ and *t*_1_ using the Wilcoxon matched pairs test for non-parametric datasets. We used Spearman’s correlation to investigate the possible association between Silencing score and Attentional Effort and Mindfulness scores.

### MRI Data Acquisition

Imaging data were acquired using a Siemens 1.5-T MAGNETOM Avanto (Siemens, Erlangen, Germany) whole body scanner equipped with a 12-element designed Head Matrix coil, as part of the standard system configuration. Diffusion-weighted images (DWIs) were acquired using an axial pulsed-gradient spin-echo echo-planar sequence (7600/103; 38 sections; section thickness, 3.0 mm with no intersection gap), with diffusion-encoding gradients applied in 12 non-collinear directions (b factor 0 and 1000 s/mm^2^; number of acquired signals, four). A 2D fluid-attenuated inversion recovery (FLAIR) T2-weighted scan was also used to exclude the presence of small vessel ischemic disease and other supra- or infra-tentorial brain lesions [Repetition Time (TR) = 11,460 ms, Echo Time (TE) = 102 ms, Inversion Time (TI) = 2360 ms, Field of View (FOV) = 280 × 330 mm, Number of Excitations (NEX) = 2, matrix = 248 × 320, 1.00 × 1.00 mm^2^ in-plane resolution, horizontal slices with a slice thickness of 3.0 mm and no gap].

### MRI Data Analysis

To avoid a type I error induced by the effect of WM hyperintensities on brain connectivity results, two expert radiologists managed by CCQ examined all MRIs. Participants were excluded when more than three lesions with a maximum diameter of 5 mm were detected in the subcortical or periventricular WM on axial FLAIR images (Quattrocchi et al., 2015).

### Preprocessing of Diffusion Data

All DWIs were visually inspected for artifacts and preprocessed using different tools from FDT (FMRIB Diffusion Toolbox, part of FSL (FMRIB’s Software Library v.5.0.8^1^; Smith et al., 2004). Images were corrected for eddy current distortion and head motion using a 12 parameter affine registration to the first no-diffusion-weighted volume of each participant, and the gradient directions were rotated accordingly (Leemans and Jones, 2009). Corrected images were skull-stripped using Brain Extraction Tool (Smith, 2002). Diffusion tensor images were then generated for each participant and each time point using the Diffusion Tensor Imaging ToolKit software package (DTI-TK^2^; Zhang et al., 2006). An unbiased longitudinal analysis approach was chosen for the registration of DTI data (Keihaninejad et al., 2013) using DTI-TK, which applies a registration algorithm that leverages the full diffusion tensor information to drive the registration, improving the alignment of WM structures (Wang et al., 2011). At the end of the registration procedure, each participant’s DTI data were normalized to the ICBM-152 template (Zhang et al., 2011), and FA maps were generated for each participant using the normalized tensor images (for details on the DTI data analysis, see Piervincenzi et al., 2017). FA data from each participant were further analyzed using selected modules of the Tract-Based Spatial Statistics (Smith et al., 2006) toolbox, available in FSL. The mean FA image was created and thinned to create a mean FA skeleton, which represents the centers of all tracts common to the group. Each participant’s FA image was then projected onto this common skeleton to minimize any residual misalignment of tracts.

To investigate the potential association between Silencing and training-induced DTI effects, a voxelwise correlation analysis was carried out between ΔSilencing (*t*_1_–*t*_0_) and ΔFA maps (*t*_1_–*t*_0_) using permutation-based non-parametric statistics via the FSL *randomize* tool (Nichols and Holmes, 2002) with 5,000 permutations. Resulting statistical maps were thresholded using FDR *q* = 0.05. Correlation analysis was performed inside the mask of previously reported longitudinal FA changes (for details on DTI analysis, see Piervincenzi et al., 2017).

## Results

### QMT Elicitation of Silence and Mind-Wandering Content

**FIGURE 1:**
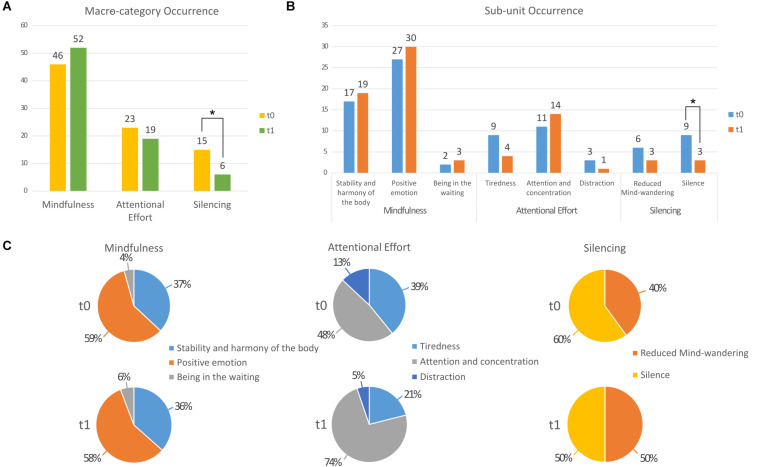
Bar graph presenting results of the occurrence analysis comparing *t*_0_ and *t*_1_ **(A)** for each macro-category and **(B)** for each sub-unit. **(C)** Pie charts presenting the score distribution within each category at *t*_0_ and at *t*_1_. *p < 0.05.

Following a session of QMT (at *t*_0_), 36% of the participants reported experiences related to Silence and 27% reported Reduced Mind-wandering. In addition, 95% of the participants reported experiences related to Mindfulness and 63% to Attentional Effort. After 6 weeks of training (*t*_1_), 9% of the participants reported experiences related to Silence and 13% to Reduced Mind-wandering. Also at *t*_1_, 95% of participants reported experiences related to Mindfulness and 72% to Attentional Effort. Thus, content in the Silencing macro-category significantly decreased between *t*_0_ and *t*_1_ (*p* < 0.04; see Figure 1A). Changes in the other macro-categories were not statistically significant (all *p* > 0.23).

When we analyzed the two sub-units of the Silencing macro-category, we observed that both Reduced mind-wandering and Silence were higher at *t*_0_ (6 and 9, respectively) than at *t*_1_ (both 3). However, only the decrease in the Silence sub-unit was significant (*p* < 0.03; Figure 1B).

The Attention and concentration sub-unit increased from 48 to 74% of the responses in the Attentional Effort macro-category, while those of Tiredness and Distraction both decreased, from 39 to 21% and from 13 to 5%, respectively, (see Figure 1C).

### Correlation Between Silencing and DTI Changes

**FIGURE 2:**
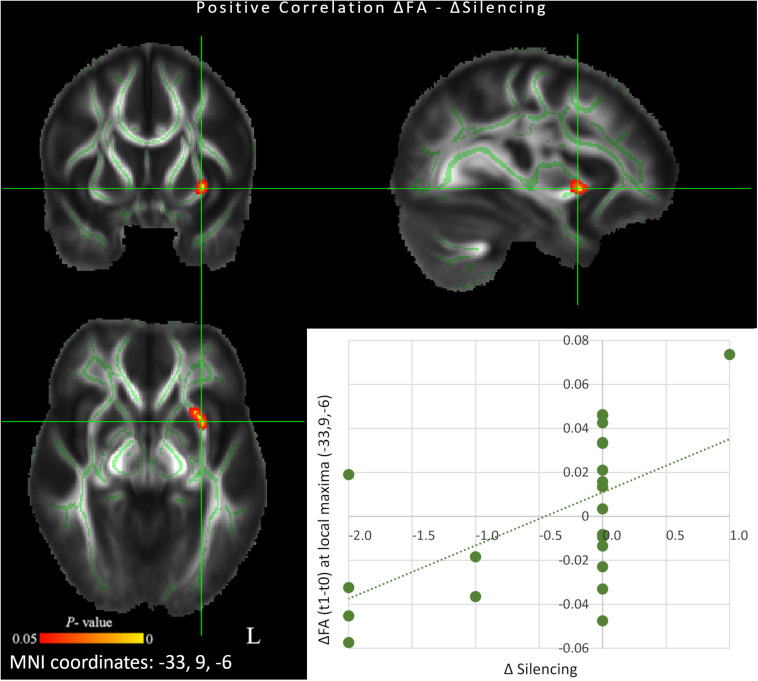
Scatterplot and results of voxel-wise correlation analysis between longitudinal changes in fractional anisotropy (FA) maps and concomitant changes in Silencing score (red-yellow color) between *t*_0_ and *t*_1_ (*p* < 0.05 FDR corrected). Only clusters showing a spatial extent of at least 30 voxels are reported. The study-specific FA skeleton, representing the centers of principal white matter tracts, is displayed in green, overlaid on the mean FA map. Anatomical localizations of peak MNI coordinates (mm) were established according to the JHU White-Matter Tractography and the JHU ICBM-DTI-81 White-Matter Labels atlases.

We observed a significant correlation between changes in Silencing and changes in FA maps following 6 weeks of QMT (*p* < 0.05 FDR corrected). More specifically, a positive correlation was found between change in Silencing and regions showing longitudinal increments of FA in left uncinate fasciculus (MNI peak coordinates: *x* = −33, *y* = 9, *z* = −6; *t* = 2.65; *r* = 0.57, *p* < 0.01) (Figure 2). Thus, greater FA increase was associated with a higher number of reports related to the experience of silence.

### Correlations Between Silencing, Attentional Effort, and Mindfulness

**FIGURE 3:**
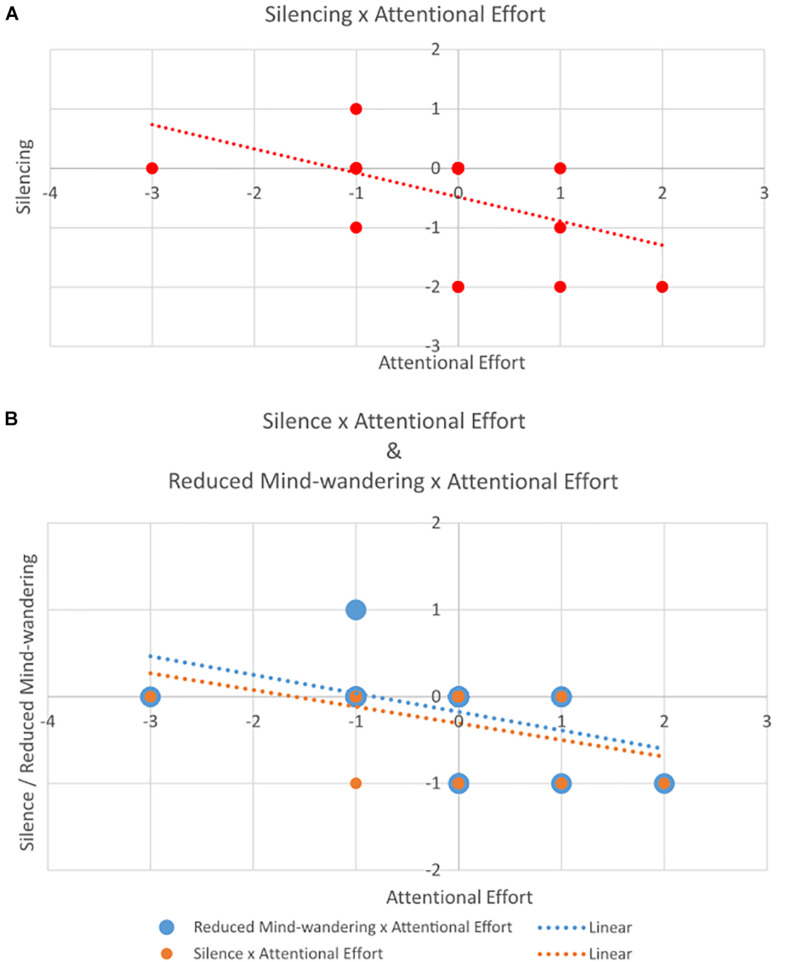
Scatterplot of correlation between change in **(A)** Silencing (*y*-axis) and Attentional Effort (*x*-axis); and **(B)** Reduced Mind-wandering and Silence (both on *y*-axis) and Attentional Effort (*x*-axis).

A significant negative correlation between change (*t*_1_–*t*_0_) in Silencing and change in Attentional Effort (*r* = −0.47, *p* < 0.05) suggested that participants who show greater change in Attentional Effort exhibit less change in Silencing, and vice versa (Figure 3A). All other correlations were non-significant (all *p* > 0.30).

We observed a strong significant positive correlation between Reduced Mind-wandering and Silence (components of the Silencing category) (*r* = 0.72, *p* < 0.001), a significant negative correlation between Attentional Effort and Reduced Mind-wandering (*r* = −0.49, *p* < 0.05), and a correlation that approached significance between Attentional Effort and Silence (*r* = -0.40, *p* = 0.058) (Figure 3B). All other correlations were non-significant (all *p* > 0.21).

We also observed an increase in co-occurrence of Positive Emotion and Attention and Concentration (from 3 to 8; c-coefficient: from 0.09 to 0.17) and a contextual decrease of Positive Emotion and Silence (from 6 to 1; c-coefficient: from 0.20 to 0.02).

## Discussion

The aim of the current study was to study the potential connection between silence-related experience during the practice of QMT and microstructural changes previously reported in Piervincenzi et al. (2017). While self-report techniques have their limitations (Rappert et al., 2017), they offer the possibility to capture the subjective dimension intrinsic to the personal experience, and thus a deeper understanding of the relationship between the different constructs, disentangling the subtle differences in the relationships among silence, attentional effort, and mindfulness.

Our results showed that 36% of participants reported experiences related to silence and 27% reported reduced mind-wandering during the first session of QMT practice. After 6 weeks of training, only 9% of the participants reported experiences related to silence and 13% reported reduced mind-wandering during practice execution. Hence, contrary to our expectation, participants reported fewer experiences related to silence and reduced mind-wandering after the 6 weeks period of daily training. It is possible that the participants experienced a sort of “normalization” or habituation to silence-related experience, reducing its salience. Namely, the same experiences of silence and reduced mind-wandering might have felt stronger and more salient to participants when they were new to QMT than when after 6 weeks of practice. In support of this interpretation, *t*_0_ reports were more complex and seemed aimed at explaining the experience of reduced thoughts (e.g., “strange absence of thoughts, more silence,” “no thoughts”), while *t*_1_ reports were simpler and more direct (e.g., “silence,” “quietness”) – as if participants had become more familiar with this kind of experience. Of course, “silence” or “quietness” might simply designate personally significant labels for more complex experiences. In this context, we note Varela’s (1996) proposition that greater integration of self-report techniques in empirical research requires that participants receive adequate training to increase their ability to report accurately. This is in line with the Tibetan tradition of denoting meditation practice with the term *gom*, which literally means *to familiarize with* (Saggar et al., 2012).

In line with our hypothesis, silence-related experiences were positively correlated with changes in the uncinate fasciculus. In humans, the uncinate fasciculus is engaged in tasks that involve naming, single word comprehension, response inhibition, face processing, and monitoring of outcomes (Arnodio and Frith, 2006; Catani et al., 2013). Disconnection of the uncinate fasciculus, on the other hand, causes impairment of object-reward association learning, and reduced performance in memory tasks involving temporally complex visual information (Gaffan and Wilson, 2008). The bidirectionality of information flow in the uncinate fasciculus allows orbitofrontal cortex-based reward and punishment history to rapidly modulate temporal lobe-based mnemonic representations (Von Der Heide et al., 2013). In accordance, while disruption of uncinate fasciculus activity might cause memory and learning deficits, enhancing its activity through training may foster the ability to evaluate past experiences, especially those related to reward and punishment (Von Der Heide et al., 2013). This, in turn, might result in more effective monitoring and management of ongoing performance, as participants in this and previous studies have reported increased attention and concentration (Ben-Soussan et al., 2017), possibly mediated by the silence required to correctly perform QMT.

Given that there are a number of functions associated with the uncinate fasciculus, one should keep in mind that the observed changes might be due to other effects of QMT training besides the construct of silence. For example, the reported changes in uncinate fasciculus might be ascribed to associative learning (Thomas et al., 2012) particularly that involving visual-motor associations (Thomas et al., 2015) to perform the QMT. In fact, the subjective experience of silence could reflect reduced cognitive processing associated with the transition from early cognitive to middle associative motor learning stages to a later autonomous stage. Thus, as one of our reviewers has suggested, the current changes in uncinate fasciculus correlated with subjective experience of silence, is so because both are reflective of motor learning. This latter view is of interest to mindfulness theory as it suggests that performing repetitive sequenced movements can establish mindfulness states and provide mindfulness training benefits after learning of the sequenced movements has progressed to the later stages of motor learning.

The current results also showed a negative correlation between the Silencing and Attentional Effort macro-categories. Moreover, the Silence and Reduced Mind-wandering sub-units showed a strong positive correlation between them, and were both negatively correlated with Attentional Effort, but not with the Mindfulness category.

In this study, we explored for the first time self-reported subjective phenomenological experiences of silence during QMT practice and their correlations with changes in structural brain connectivity. However, it is not clear what is the mechanism underling these changes. Future studies should examine the causal relationship between QMT experience-related reports of silence and changes in FA by means of more specific assessments related to silence, as well as comparing it to additional training groups. The uncinate fasciculus matures later and more slowly than other brain fiber connections, and might continue developing beyond the age of 30 years (Lebel et al., 2008). This might enable greater structural plasticity in response to a variety of internal or external (De Fano et al., 2019) environmental influences (Markowitsch and Staniloiu, 2011), such as induced internal and external silence.

## Limitations and Conclusion

The lack of a control group is one of the main limitations of this study. However, neural, neuronal, and cognitive changes related to QMT have been compared to different control groups in the past, controlling for both cognitive and motor load, demonstrating its specificity (see Ben-Soussan et al., 2013, 2014b; Venditti et al., 2015; Paoletti et al., 2017).

More in detail, QMT related changes have been studied by comparing groups performing QMT with active control groups performing verbal or motor tasks (Ben-Soussan et al., 2013). This study demonstrated that cognitive and neurophysiological changes can be specifically induced by QMT practice (Ben-Soussan et al., 2013). Moreover, QMT’s impact on emotional regulation has been studied comparing two groups of meditators, one performing a breathing meditation + QMT and the other one performing only the breathing meditation (Paoletti et al., 2017). In this case, increased affect balance was attributed to the group which also performed the QMT practice, while self-efficacy increased in both groups.

For these reasons, in our opinion, the lack of a control group in the present study represents a limitation but taking in consideration (1) the observed reliable specificity of QMT-related cognitive, neurophysiological and emotional modulation, (2) the results of other studies showing improvements in self-efficacy, emotional and cognitive modulations following mindfulness or meditative practices (Creswell et al., 2007; Garland et al., 2009, 2011; Wendt et al., 2015), we can associate the here presented results about silence reports and their correlation with uncinate fasciculus connectivity to the prolonged performance of QMT as a meditative practice.

Moreover, several studies have already demonstrated the longitudinal reliability of DTI measures, including previous learning studies where control groups did not show FA changes (Scholz et al., 2009; Taubert et al., 2010). Nevertheless, despite demonstrations from previous work, the present findings are limited by the lack of a control condition. Another possible interpretation which cannot be excluded by the present work, involves the expectation due to receiving the intervention.

It is important to underline that the content of this manuscript goes beyond the main aim pursued by the original longitudinal study, of studying the cognitive, emotional, and physical experiences related to QMT performance. The exploration of silence-related experience to QMT practice was not part of the experimental design, and participants have never been directly asked or cued to mention it. Nonetheless, it emerged spontaneously from first-person reports, suggesting that silence could represent a particularly relevant component of this meditative practice.

This study provided interesting insights, but further investigations specifically designed to assess the impact of the experience of silence in meditative sensorimotor trainings and their neurophysiological correlates are required.

## Data Availability Statement

The raw data supporting the conclusions of this article will be made available by the authors, without undue reservation.

## Ethics Statement

The studies involving human participants were reviewed and approved by the Ethical Committee of the Università Campus Bio-Medico di Roma. The patients/participants provided their written informed consent to participate in this study.

## Author Contributions

TB-S and FC designed the research. CQ performed the research. FM, AD, and FA analyzed the first-person reports. CP and FC analyzed the DTI data and DTI correlations. TB-S, FM, CP, JG, and FC wrote the manuscript. AD, FA, and CQ contributed to the writing process. All authors contributed to the article and approved the submitted version.

## Conflict of Interest

The authors declare that the research was conducted in the absence of any commercial or financial relationships that could be construed as a potential conflict of interest. The handling Editor declared a shared affiliation, though no other collaboration, with several of the authors CP and FC the at time of the review.

## Footnotes

1 https://www.fmrib.ox.ac.uk/fsl/

2 https://dti-tk.sourceforge.net/pmwiki/pmwiki.php
